# Polymorphisms of *TGFBR1*, *TLR4* are associated with prognosis of gastric cancer in a Chinese population

**DOI:** 10.1186/s12935-018-0682-0

**Published:** 2018-11-20

**Authors:** Bangshun He, Tao Xu, Bei Pan, Yuqin Pan, Xuhong Wang, Jingwu Dong, Huiling Sun, Xueni Xu, Xiangxiang Liu, Shukui Wang

**Affiliations:** 1 General Clinical Research Center, Nanjing First Hospital, Nanjing Medical University, 68 Changle Road, Nanjing, 210006 China; 2 Helicobacter pylori Research Key Laboratory, Nanjing Medical University, Nanjing, 210000 China; 3 Medical College, Southeast University, Nanjing, 210000 China; 4 Digestive Department, Xuyi People’s Hospital, Huaian, 211700 China

**Keywords:** *IL*-*16*, *TGFBR1*, *TLR4*, Polymorphism, Gastric cancer, Susceptibility, Prognosis

## Abstract

**Background:**

*Helicobacter pylori* (*H. pylori*)-induced gastric cancer is an intricate progression of immune response against *H. pylori* infection. IL-16, TGF-β1 and TLR4 pathways were the mediators involved in the immune response. We hypothesized that genetic variations in genes of these pathways have potential susceptibility to gastric cancer risk, and predict clinical outcomes of patients.

**Methods:**

To investigate the susceptibility and prognostic value of genetic variations of *IL*-*16*, *TGFBR1* and *TLR4* pathways to gastric cancer, we performed a case–control study combined a retrospective study in a Chinese population. Genotyping for all polymorphisms was based on the Sequenom’s MassARRAY platform, and *H. pylori* infection was determined by using an immunogold testing kit.

**Results:**

We found rs10512263 CC genotype was found to be a decreased risk of gastric cancer (CC vs. TT: adjusted OR = 0.54, 95% CI 0.31–0.97); however, rs334348 GG genotype was associated with increased risk of gastric cancer (GG vs. AA: adjusted OR = 1.51, 95% CI 1.05–2.18). We found that carriers harboring rs1927911 A allele (GA/AA) or rs10512263 C allele (CT/CC) have unfavorable survival time than none carriers (rs1927911: GA/AA vs. GG: adjusted HR = 1.27, 95% CI 1.00–1.63; rs10512263: CT/CC vs. TT: adjusted HR = 1.29, 95% CI 1.02–1.63) and that individuals harboring both two minor alleles (rs1927911 GA/AA and rs10512263 CT/CC) suffered a significant unfavorable survival (adjusted HR = 1.64, 95% CI 1.17–2.31).

**Conclusion:**

In short, we concluded that two polymorphisms (rs334348, rs10512263) in *TGFBR1* were associated with risk of gastric cancer, and that *TLR4* rs1927911 and *TGFBR1* rs10512263 were associated with clinical outcomes of gastric cancer patients.

**Electronic supplementary material:**

The online version of this article (10.1186/s12935-018-0682-0) contains supplementary material, which is available to authorized users.

## Background

Gastric cancer is the fifth most common cancer worldwide and ranks third cause of cancer related mortality [1]. Almost over half of new diagnosed cases are from eastern Asian, predominantly in China [2]. Gastric cancer is a multifactorial disease with multistep etiology. Epidemiological studies have demonstrated that interaction of environmental factors, such as *Helicobacter pylori* (*H. pylori*) infection, excessive salt intake, alcohol drinking and tobacco smoking, and genetic background was regarded as risk of gastric cancer.

For environmental factors, *H. pylori* causing chronic inflammation has been verified as a key factor involved in gastric carcinogenesis. Moreover, for genetic background, polymorphisms in immune-related genes, such as *IL*-*1B*, *IL*-*1RN*, *IL*-*10*, could affect their expression and were suggested as risk factors of gastric cancer [3, 4]. In addition, we previously reported genetic polymorphisms in the promoter of *IL*-*1B/IL*-*1RN* were the risk of gastric cancer [5, 6]. Of immune-related genes, IL-16 is a pro-inflammatory cytokine that has a variety of biological functions, playing role in the development and homeostasis of the immune system [7], and stimulating the secretion of tumor-associated inflammatory cytokines including TNF-α, IL-1β, IL-6, and IL-15 [8]. In addition, polymorphisms in *IL*-*16* were investigated to be risk of various cancers, including gastric cancer, and the diagnostic and prognostic value of serum IL-16 levels for patients with gastric cancer was also reported [9]. Transforming growth factor beta-1 (TGF-β1), a multifunctional cytokine, combined it’s receptor (*TGFBR1*) plays biphasic role in carcinogenesis that, in early stages of cancer, it acts as a tumor suppressor by inhibiting cellular proliferation or by promoting cellular differentiation and apoptosis; in later stages of cancer, however, it turns to be a tumor promoter by stimulating angiogenesis and cell motility, suppressing immune response, and increasing progressive invasion and metastasis [10–12]. Moreover, serum TGF-β1 levels implicating a predictive and prognostic value for patients with gastric cancer [13, 14] may indicate polymorphisms in genes of TGF-β1 pathway including TGFBR1 could influence the risk and clinical progression of gastric cancer [15–17]. In the progression of *H. pylori* infection, toll-like receptors (TLRs), a group of membrane-bound receptors proteins, play a pivotal role in innate immune response and provide first line of host defense. Among TLRs, TLR-4 is the main receptor of lipopolysaccharide (LPS) and plays a role in initiating the inflammatory response of *H. pylori* infection. After binding of microbial ligands, a dysregulation of TLR signalling may contribute to an unbalanced ratio between pro- and anti-inflammatory cytokines, resulting in increasing higher risk of developing gastric cancer [18]. Similarly, polymorphisms in *TLR4* has been implicated as risk factors for gastric cancer [18]; however, the conclusion of susceptibility of these polymorphisms to gastric cancer risk remains elusive [19–21].

Immune response triggered by *H. pylori* infection, including host adaptive immune response (such as IL-1b, TNF-a, IL-10, IL-16) and innate immune response (such as TLR4), is an intricate progression, which is responsible for clinical outcomes of individuals with *H. pylori* infection. Thus, polymorphisms occurring in immune genes could serves as possible susceptibility factors to the development of gastric cancer and have a predictive value for gastric cancer clinical outcome. Here, we conducted a case–control study to assess the susceptibility of polymorphisms in *IL*-*16*, *TGFBR1* and *TLR4* to risk of gastric cancer in a Chinese population, and the prognostic value of the polymorphisms was also evaluated by a retrospective study.

## Materials and methods

### Study population

For the case–controls study, we recruited 479 patents histologically diagnosed as gastric cancer and 483 age- and sex-matched healthy controls who came to the hospital for routine physical examination. The demographic features of participants were collected via a questionnaire or by reviewing patients’ medical records. The TNM stages were classified according to American Joint Commission for Cancer Staging in 2002 (the sixth edition). For retrospective study, we traced survival state of all patients through on-site interview, direct calling or medical chart review, and finally, a total of 460 patients were followed up to 5 years. The protocol of this study was approved by the Institutional Review Board of the Nanjing First Hospital, and written informed consents were obtained from all participants.

### DNA extraction and genotyping

We retrieved the potential genetic variations in *IL*-*16*, *TGF*-*BR1* and *TLR4* from the National Center for Biotechnology Information dbSNP database (http://www.ncbi.nlm.nih.gov/projects/SNP), and then the genetic variations were selected followed the following criteria: (1) the minor allele frequency (MAF) is not less than 5% in Han Chinese population; (2) with position in exons, promoter region, 5′ untranslated regions (UTR) or 3′ UTR; and (3) published results shown to be associated with any cancer risk. For those polymorphisms in intron if meet the criterion (3) were also included. Finally, a total of 11 polymorphisms were selected (Additional file 1: Table S1).

The DNA extraction and genotyping was performed as we previously described [22]. A GoldMag-Mini Whole Blood Genomic DNA Purification Kit (GoldMag Co. Ltd. Xi’an, China) was used for DNA extraction, and then the genotyping was performed on the SequenomMassARRAY platform.

### *H. pylori* infection detection

To identify the *H. pylori* infection, the serum of all participants were collected to detect *H. pylori* antibody by using a *H. pylori* immunogold testing kit (KangmeiTianhong Biotech Co., Ltd, Beijing, China).

#### Statistical analysis

The difference of demographic features of the two groups was assessed by *t* test or χ^2^ test. For the distribution of genotypes, a goodness of fit Chi square test was adopted to test the Hardy–Weinberg equilibrium (HWE) in the control group, and then, the susceptibility of polymorphisms to gastric cancer risk was expressed with odds ratios (ORs) and 95% confidence intervals (CIs). Subgroups analyze was conducted if there was a significant association of the polymorphism to gastric cancer risk. The risk of polymorphisms was calculated by using a logistic regression model based on SAS v9.1 (SAS Institute, Cary, NC, USA). The hazard ratios (HRs) of genotypes to survival time of patients were calculated by Cox regression analysis with SPSS 11.0 (SPSS, Chicago, IL, USA). The p value < 0.05 was considered statistically significant difference.

## Result

### Characteristics of the study population

The health controls and patients were matched for age (p = 0.748) and gender (p = 0.881). There were significant differences between the two groups with respect to the frequency of *H. pylori* infection (p = 0.039), cigarette smoking (p < 0.001) and alcohol consumption (p < 0.001), summarized in Additional file 1: Table S2. The observed frequencies of all tested genotypes in controls did not deviate from HWE (shown in Additional file 1: Table S1).

### Association between polymorphisms and risk of gastric cancer

Two polymorphisms in *TGFBR1* were observed to be potentially associated with risk of gastric cancer. rs10512263 CC genotype was found to be a decreased risk of gastric cancer (CC vs. TT: adjusted OR = 0.54, 95% CI 0.31–0.97, p = 0.039); however, rs334348 GG genotype was associated with increased risk of gastric cancer (GG vs. AA: adjusted OR = 1.51, 95% CI 1.05–2.18, p = 0.028), shown in Table 1.

**Table 1:** Association between polymorphisms and risk of gastric cancer

Polymorphism	Genotype	Cases, n (%)	Controls, n (%)	OR (95% CI)	OR (95% CI)^a^	*p* value
*IL*-*16* rs4072111	CC	334 (69.73)	345 (71.43)	Reference	Reference	
	TC	126 (26.30)	122 (25.26)	1.07 (0.80,1.43)	1.01 (0.75,1.36)	0.970
	TT	19 (3.97)	16 (3.31)	1.23 (0.62,2.43)	1.20 (0.60,2.41)	0.600
	TC/TT	145 (30.27)	138 (28.57)	1.09 (0.82,1.43)	1.02 (0.77,1.36)	0.870
	Additive model			1.08 (0.86,1.37)	1.04 (0.82,1.32)	0.755
rs4778889	TT	267 (55.74)	266 (55.07)	Reference	Reference	
	CT	182 (38.00)	192 (39.75)	0.94 (0.73,1.23)	0.92 (0.70,1.20)	0.524
	CC	30 (6.26)	25 (5.18)	1.20 (0.68,2.09)	1.19 (0.68,2.10)	0.542
	CT/CC	212 (44.26)	217 (44.93)	0.97 (0.76,1.26)	0.95 (0.73,0.23)	0.688
	Additive model			1.01 (0.82,1.25)	1.00 (0.81,1.23)	0.965
rs859	TT	129 (26.93)	124 (25.67)	Reference	Reference	
	CT	235 (49.06)	248 (51.35)	0.91 (0.67,1.24)	0.88 (0.64,1.20)	0.406
	CC	115 (24.01)	111 (22.98)	1.00 (0.70,1.43)	0.98 (0.68,1.41)	0.899
	CT/CC	350 (73.07)	359 (74.33)	0.94 (0.70,1.25)	0.92 (0.68,1.23)	0.551
	Additive model			1.00 (0.83,1.19)	0.98 (0.82,1.18)	0.859
rs11556218	TT	306 (63.88)	308 (63.77)	Reference	Reference	
	GT	151 (31.52)	157 (32.51)	1.97 (0.74,1.27)	0.93 (0.71,1.23)	0.628
	GG	22 (4.59)	18 (3.73)	1.23 (0.65,2.34)	1.29 (0.68,2.48)	0.439
	GT/GG	173 (36.12)	175 (36.23)	1.00 (0.77,1.29)	0.97 (0.94,1.27)	0.820
	Additive model			1.02 (0.82,1.28)	1.01 (0.81,1.27)	0.913
rs1131445	TT	221 (46.14)	210 (43.48)	Reference	Reference	
	CT	211 (44.05)	222 (45.96)	0.90 (0.69,1.18)	0.94 (0.72,1.23)	0.655
	CC	47 (9.81)	51 (10.56)	0.88 (0.57,1.36)	0.93 (0.59,1.46)	0.748
	CT/CC	258 (53.86)	273 (56.52)	0.90 (0.70,1.16)	0.93 (0.72,1.21)	0.592
	Additive model			0.92 (0.76,1.12)	0.95 (0.78,1.16)	0.618
*TLR4* rs10759932	TT	240 (50.10)	251 (51.97)	Reference	Reference	
	TC	191 (39.87)	196 (40.58)	1.02 (0.78,1.33)	1.05 (0.80,1.38)	0.733
	CC	48 (10.02)	36 (7.45)	1.39 (0.87,2.22)	1.38 (0.86,2.23)	0.184
	TC/CC	239 (49.90)	232 (48.03)	1.08 (0.84,1.39)	1.10 (0.85,1.42)	0.481
	Additive model			1.11 (0.91,1.35)	1.12 (0.92,1.36)	0.275
rs1927911	GG	171 (35.70)	175 (36.23)	Reference	Reference	
	GA	226 (47.18)	226 (46.79)	1.02 (0.77,1.35)	1.04 (0.78,1.38)	0.801
	AA	82 (17.12)	82 (16.98)	1.02 (0.71,1.48)	0.99 (0.68,1.45)	0.967
	GA/AA	308 (64.30)	308 (63.77)	1.02 (0.79,1.33)	1.03 (0.79,1.34)	0.844
	Additive model			1.01 (0.85,1.21)	1.01 (0.84,1.21)	0.930
rs11536889	GG	303 (63.26)	293 (60.66)	Reference	Reference	
	CG	156 (32.57)	166 (34.37)	0.91 (0.69,1.19)	0.91 (0.69,1.19)	0.477
	CC	20 (4.18)	24 (4.97)	0.81 (0.44,1.49)	0.76 (0.40,1.43)	0.392
	CG/CC	176 (36.74)	190 (39.34)	0.90 (0.69,1.16)	0.89 (0.69,1.16)	0.402
	Additive model			0.90 (0.73,1.13)	0.90 (0.72,1.12)	0.355
*TGF*-*BR1* rs6478974	TT	219 (45.72)	194 (40.17)	Reference	Reference	
	AT	204 (42.59)	220 (45.55)	0.82 (0.63,1.08)	0.80 (0.61,1.06)	0.118
	AA	56 (11.69)	69 (14.29)	0.72 (0.48,1.08)	0.68 (0.45,1.02)	0.063
	AT/AA	260 (54.28)	289 (59.83)	0.80 (0.62,1.03)	0.78 (0.60,1.01)	0.055
	Additive model			0.84 (0.70,1.01)	*0.82 (0.68,0.99)*	*0.038*
rs334348	AA	143 (29.85)	158 (32.71)	Reference	Reference	
	AG	221 (46.14)	240 (49.69)	1.02 (0.76,1.36)	1.05 (0.78,1.42)	0.730
	GG	115 (24.01)	85 (17.60)	1.50 (1.04,2.14)	*1.51 (1.05,2.18)*	*0.028*
	AG/GG	336 (70.15)	325 (69.29)	1.14 (0.87,1.50)	1.17 (0.89,1.55)	0.263
	Additive model			1.20 (1.01,1.43)	*1.22 (1.02,1.46)*	*0.032*
rs10512263	TT	279 (58.25)	262 (54.24)	Reference	Reference	
	CT	178 (37.16)	187 (38.72)	0.89 (0.69,1.17)	0.87 (0.66,1.14)	0.297
	CC	22 (4.59)	34 (7.04)	0.61 (0.35,1.07)	*0.54 (0.31,0.97)*	*0.039*
	CT/CC	200 (41.75)	221 (45.76)	0.85 (0.66,1.10)	0.82 (0.63,1.06)	0.127
	Additive model			0.84 (0.68,1.03)	*0.81 (0.65,1.00)*	*0.047*

Italic represents any values with p < 0.05

*OR* odds ratio

^a^Adjusted for age, gender, smoking, drinking, and *H. pylori* infection status

Stratified analysis by age, gender, *H. pylori* infection status, tumor stage and tumor site revealed that the significant association of rs10512263 to risk of gastric cancer was maintained in the subgroup of male, and subgroup of individuals with older age, shown in Table 2. In the stratification analysis by pathologic characteristics, we observed that the significant association of rs334348 to risk of gastric cancer was maintained in the subgroup of patients with clinical stage T1–T2. In addition, although no significant association was found, aboundary significant of two polymorphisms to risk of gastric cancer was observed in subgroup of clinical stage T1–T2 and in subgroup of non-cardiac, shown in Table 3.

**Table 2:** Stratification analyses the association between polymorphisms in *TGF*-*BR1* and gastric cancer risk

Genotype	Age						Gender						*H. pylori* infection					
	≤ 64			> 64			Male			Female			Positive			Negative		
	Ca/Co	OR (95% CI)^a^	*p* value	Ca/Co	OR (95% CI)^a^	*p* value	Ca/Co	OR (95% CI)^a^	*p* value	Ca/Co	OR (95% CI)^a^	*p* value	Ca/Co	OR (95% CI)^a^	*p* value	Ca/Co	OR (95% CI)^a^	*p* value
rs6478974																		
TT	100/87	Reference		119/107	Reference		156/134	Reference		63/60	Reference		120/91	Reference		99/103	Reference	
AT	101/113	0.76 (0.51,1.13)	0.175	103/107	0.85 (0.58,1.25)	0.412	159/170	0.80 (0.58,1.10)	0.164	45/50	0.81 (0.46,.41)	0.452	108/107	0.76 (0.52,1.12)	0.160	96/113	0.86 (0.58,1.28)	0.457
AA	28/30	0.76 (0.41,1.40)	0.374	28/39	0.61 (0.34,1.07)	0.082	38/54	*0.55 (0.33,0.90)*	*0.017*	18/15	1.19 (0.55,2.58)	0.663	33/33	0.73 (0.41,1.28)	0.272	23/36	0.62 (0.34,1.14)	0.127
AT/AA	129/143	0.76 (0.52,1.11)	0.156	131/146	0.80 (0.56,1.14)	0.206	197/224	*0.74 (0.54,1.00)*	*0.049*	63/65	0.89 (0.54,1.48)	0.658	141/140	0.75 (0.52,1.08)	0.125	119/149	0.81 (0.55,1.17)	0.260
Additive model		0.83 (0.63,1.10)	0.199		0.81 (0.62,1.04)	0.100		*0.76 (0.61,0.95)*	*0.016*		1.01 (0.70,1.44)	0.975		0.83 (0.64,1.08)	0.157		0.81 (0.62,1.07)	0.135
rs334348																		
AA	72/74	Reference		71/84	Reference		108/121	Reference		35/37	Reference		77/83	Reference		66/75	Reference	
AG	105/122	0.95 (0.62,1.47)	0.832	116/118	1.16 (0.77,1.76)	0.478	168/173	1.15 (0.81,1.62)	0.143	53/67	0.85 (0.46,1.54)	0.583	124/100	1.43 (0.94,2.17)	0.093	97/140	0.79 (0.51,1.21)	0.278
GG	52/34	1.57 (0.90,2.73)	0.114	63/51	1.44 (0.88,2.37)	0.146	77/64	1.37 (0.89,2.11)	0.149	38/21	1.97 (0.94,4.11)	0.072	60/48	0.37 (0.83,2.25)	0.223	55/37	1.71 (0.99,2.53)	0.054
AG/GG	157/156	1.09 (0.72,1.63)	0.694	179/169	1.26 (0.86,1.85)	0.245	245/237	1.20 (0.87,1.66)	0.269	91/88	1.10 (0.63,1.92)	0.745	184/148	1.40 (0.95,2.06)	0.088	152/177	0.98 (0.66,1.47)	0.927
Additive model		1.21 (0.93,1.59)	0.161		1.22 (0.95,1.56)	0.113		1.17 (0.95,1.45)	0.141		1.36 (0.95,1.94)	0.096		1.19 (0.93,1.53)	0.166		1.25 (0.96,1.64)	0.097
rs10512263																		
TT	130/129	Reference		149/133	Reference		202/189	Reference		77/73	Reference		150/127	Reference		129/135	Reference	
CT	88/88	0.95 (0.64,1.41)	0.814	90/99	0.80 (0.55,1.16)	0.230	136/140	0.89 (0.65,1.22)	0.470	42/47	0.82 (0.48,1.41)	0.471	98/87	0.91 (0.62,1.33)	0.610	80/100	0.83 (0.56,1.22)	0.335
CC	11/13	0.70 (0.29,1.67)	0.417	11/21	*0.44 (0.20,0.96)*	*0.040*	15/29	*0.41 (0.21,0.81)*	*0.010*	7/5	1.40 (0.42,4.63)	0.585	13/17	0.64 (0.29,1.39)	0.258	9/17	0.45 (0.19,1.10)	0.080
CT/CC	99/101	0.91 (0.62,1.33)	0.621	101/120	0.74 (0.52,1.06)	0.101	151/169	0.80 (0.59,1.09)	0.159	49/52	0.87 (0.52,1.46)	0.604	111/104	0.86 (0.60,1.23)	0.405	89/117	0.77 (0.53,1.13)	0.180
Additive model		0.88 (0.64,1.21)	0.441		*0.74 (0.55,0.99)*	*0.040*		*0.76 (0.60,0.98)*	*0.033*		0.96 (0.63,1.48)	0.864		0.85 (0.63,1.14)	0.272		0.76 (0.56,1.04)	0.083

Italic represents any values with p < 0.05

*OR* odds ratio, *Ca* case, *Co* control

^a^Adjusted for age, gender, smoking, drinking, and *H. pylori* infection status

**Table 3:** Stratification analyses the association between polymorphisms in *TGF*-*BR1* and gastric cancer by pathologic characteristics

Genotype	Co	Clinical stage						Tumor site					
		T1–T2			T3–T4			Cardiac			Non-cardiac		
		Ca	OR (95% CI)^a^	*p* value	Ca	OR (95% CI)^a^	*p* value	Ca	OR (95% CI)^a^	*p* value	Ca	OR (95% CI)^a^	*p* value
rs6478974													
TT	194	75	Reference		144	Reference		62	Reference		157	Reference	
AT	220	69	0.82 (0.55,1.21)	0.311	135	0.79 (0.58,1.08)	0.140	62	0.87 (0.58,1.31)	0.497	142	0.78 (0.57,1.05)	0.105
AA	69	15	0.54 (0.28,1.04)	0.065	41	0.76 (0.49,1.20)	0.238	14	0.61 (0.32,1.17)	0.139	42	0.72 (0.45,1.13)	0.147
AT/AA	289	84	0.75 (0.52,1.09)	0.134	176	0.79 (0.59,1.05)	0.109	76	0.80 (0.54,1.18)	0.268	184	0.77 (0.58,1.02)	0.069
Additive model			0.76 (0.57,1.01)	0.054		0.85 (0.69,1.05)	0.140		0.80 (0.60,1.07)	0.135		0.83 (0.67,1.02)	0.077
rs334348													
AA	158	45	Reference		98	Reference		40	Reference		103	Reference	
AG	240	73	1.19 (0.76,1.87)	0.442	148	1.01 (0.73,1.41)	0.937	64	1.14 (0.73,1.80)	0.565	157	1.04 (0.75,1.45)	0.825
GG	85	41	*1.73 (1.03,2.90)*	*0.039*	74	1.42 (0.94,2.13)	0.092	34	1.56 (0.91,2.68)	0.103	81	1.48 (0.99,2.21)	0.055
AG/GG	325	114	1.31 (0.87,1.99)	0.196	222	1.12 (0.82,1.52)	0.487		1.24 (0.81,1.89)	0.323		1.15 (0.85,1.57)	0.373
Additive model			*1.33 (1.02,1.73)*	*0.035*		1.17 (0.96,1.43)	0.125		1.25 (0.95,1.64)	0.108		1.21 (0.99,1.47)	0.069
rs10512263													
TT	262	99	Reference		180	Reference		82	Reference		197	Reference	
CT	187	53	0.75 (0.51,1.12)	0.161	125	0.93 (0.69,1.25)	0.620	50	0.81 (0.54,1.21)	0.300	128	0.90 (0.67,1.21)	0.479
CC	34	7	0.51 (0.21,1.24)	0.137	15	0.59 (0.31,1.13)	0.113	6	0.51 (0.20,1.28)	0.149	16	0.56 (0.29,1.07)	0.080
CT/CC	221	60	0.71 (0.49,1.05)	0.084	140	0.88 (0.66,1.17)	0.366	56	0.76 (0.51,1.12)	0.162	144	0.85 (0.64,1.13)	0.256
Additive model			0.73 (0.53,1.01)	0.054		0.85 (0.67,1.08)	0.184		0.76 (0.55,1.05)	0.093		0.83 (0.66,1.05)	0.120

Italic represents any values with p < 0.05

*OR* odds ratio, *Ca* case, *Co* control

^a^Adjusted for age, gender, smoking, drinking, and *H. pylori* infection status

#### Association between polymorphisms and clinical outcome

A retrospective study was conducted based on 460 patients with follow-up information on survival period of 5 years. We found that carriers harboring rs1927911 A allele (GA/AA) or rs10512263 C allele (CT/CC) have unfavorable survival time than none carriers (rs1927911: GA/AA vs. GG: adjusted HR = 1.27, 95% CI 1.00–1.63, p = 0.054; rs10512263: CT/CC vs. TT: adjusted HR = 1.29, 95% CI 1.02–1.63, p = 0.031), shown in Table 4.

**Table 4:** Association between polymorphism and overall survival of gastric cancer patients in co-dominant model

Genotype	Cases, n	Death, n (%)	Log-rank p-value	HR	HR (95% CI)^a^	p-value
rs4072111						
CC	322	205 (0.64)		Reference	Reference	
TC/TT	138	81 (0.59)	0.344	0.88 (0.68,1.14)	1.12 (0.86,1.45)	0.408
rs4778889						
TT	256	172 (0.67)		Reference	Reference	
CT/CC	204	114 (0.56)	0.028	0.77 (0.61,0.97)	0.84 (0.66,1.06)	0.146
rs11556218						
TT	293	192 (0.66)		Reference	Reference	
GT/GG	167	94 (0.56)	0.110	0.82 (0.64,1.05)	0.94 (0.73,1.20)	0.607
rs859						
AA	109	68 (0.62)		Reference	Reference	
GA/GG	351	218 (0.62)	0.633	1.07 (0.81,1.40)	1.03 (0.79,1.36)	0.814
rs1131445						
TT	211	127 (0.60)		Reference	Reference	
CT/CC	249	159 (0.64)	0.150	1.18 (0.94,1.50)	1.06 (0.84,1.35)	0.617
rs10759932						
TT	231	141 (0.61)		Reference	Reference	
TC/CC	229	145 (0.63)	0.563	1.07 (0.85,1.35)	1.07 (0.84,1.35)	0.588
rs1927911						
GG	165	95 (0.58)		Reference	Reference	
GA/AA	295	191 (0.65)	0.113	1.22 (0.95,1.56)	*1.27 (1.00,1.63)*	0.054
rs11536889						
GG	293	181 (0.62)		Reference	Reference	
CG/CC	167	105 (0.63)	0.957	1.01 (0.79,1.28)	0.99 (0.77,1.26)	0.924
rs6478974						
TT	212	126 (0.59)		Reference	Reference	
TA/AA	248	160 (0.65)	0.224	1.16 (0.92,1.46)	1.23 (0.98,1.56)	0.079
rs334348						
GG	110	64 (0.58)		Reference	Reference	
AG/AA	350	222 (0.63)	0.491	1.10 (0.84,1.46)	1.04 (0.79,1.38)	0.787
rs10512263						
TT	269	157 (0.58)		Reference	Reference	
CT/CC	191	129 (0.68)	0.031	*1.29 (1.02,1.63)*	*1.29 (1.02,1.63)*	*0.031*

Italic represents any values with p < 0.05

^a^Adjusted for age, sex, tumor site and TNM stage

The stratified analysis based on the age, gender, tumor site or clinical stage was also performed for the significant polymorphisms, and the result revealed that carriers with rs1927911 A allele have poor survival in subgroup of patients with age younger than 64 years old (GA/AA vs. GG: adjusted HR = 1.64, 95% CI 1.13–2.38), male (GA/AA vs. GG: adjusted HR = 1.36, 95% CI 1.03–1.81), and non-cardiac gastric cancer (GA/AA vs. GG: adjusted HR = 1.34, 95% CI 1.00–1.80), and that rs1927911 A allele carriers have poor survival in the subgroup of male (CT/CC vs. TT: adjusted HR = 1.43, 95% CI 1.09–1.87), patients in clinical stage T1–T2 (CT/CC vs. TT: adjusted HR = 2.54, 95% CI 1.38–4.69), and non-cardiac gastric cancer (NCGC) (CT/CC vs. TT: adjusted HR = 1.36, 95% CI 1.02–1.80), shown in Table 5.

**Table 5:** Subgroup analyses of association between polymorphisms and survival in co-dominant model

Group	Case, n	Death, n (%)	rs1927911			rs10512263		
			GA/AA: GG	HR (95% CI)^a^	p-value	CT/CC: TT	HR (95% CI)^a^	P-value
Age								
< 64	224	130 (0.58)	*142/82*	*1.64 (1.13,2.38)*	*0.009*	97/127	1.34 (0.95,1.90)	0.099
≥ 64	236	156 (0.66)	153/83	1.04 (0.75,1.45)	0.817	94/142	1.19 (0.86,1.64)	0.286
Gender								
Male	338	214 (0.63)	*216/122*	*1.36 (1.03,1.81)*	*0.033*	*145/193*	*1.43 (1.09,1.87)*	*0.010*
Female	122	72 (0.64)	79/43	1.08 (0.65,1.80)	0.754	46/76	1.26 (0.76,2.08)	0.365
Clinical stage								
T1–T2	159	42 (0.26)	102/57	1.36 (0.70,2.66)	0.367	*60/99*	*2.61 (1.40,4.86)*	*0.003*
T3–T4	301	244 (0.81)	193/108	1.21 (0.93,1.58)	0.160	131/170	1.04 (0.80,1.34)	0.784
Tumor site								
Cardiac	132	87 (0.66)	91/41	1.07 (0.67,1.71)	0.768	54/78	1.47 (0.94,2.31)	0.094
Non-cardiac	328	199 (0.61)	*204/124*	*1.34 (1.00,1.80)*	*0.050*	*137/191*	*1.36 (1.02,1.80)*	*0.034*

Italic represents any values with p < 0.05

^a^Adjusted for age, sex, tumor site and TNM stage

To identify the impact of the co-occurrence of rs1927911 and rs10512263 on overall survival, we analyzed the association between locus–locus interaction and overall survival, and the result shown that individuals harboring both two minor alleles (rs1927911GA/AA and rs10512263CT/CC) suffered a significant unfavorable survival (adjusted HR = 1.64, 95% CI 1.17–2.31), shown in Table 6.

**Table 6:** Locus–locus interactions between rs1927911 and rs10512263 and survival

rs1927911	rs10512263	Cases, n	Death, n (%)	Log-rank p value	HR (95% CI)^a^	p-value
GG	TT	100	53 (53.00)	0.018	Reference	
GG	CT/CC	65	42 (64.42)		1.18 (0.79,1.03)	0.421
GA/AA	TT	169	104 (61.54)		1.20 (0.86,1.67)	0.279
GA/AA	CT/CC	126	87 (69.05)		*1.64 (1.17,2.31)*	*0.005*

Italic represents any values with p < 0.05

^a^Adjusted for age, sex, tumor site and TNM stage

## Discussion

This case–control study combined retrospective study observed that two polymorphisms (rs334348, rs10512263) in *TGFBR1* were associated with risk of gastric cancer, and that rs1927911and rs10512263 were associated with survival of gastric cancer patients.

*TGFBR1* rs6478974 is a genetic variation in intron 1, it was previously reported to be associated with microRNAs expression and involved in carcinogenesis [23]. In addition, the significant association of rs6478974 to gastric cancer risk was also reported [15]; however, in this study, we observed such a significant association in the subgroup of male but for all participants, indicating male carrying rs6478974 polymorphisms have higher gastric cancer risk than female. Another polymorphism rs10512263 locating intron 1 of *TGBR1* was observed as a susceptibility of gastric cancer in this study; however, an opposite result was also reported [15]. It is noted that, in the subgroup analysis, we observed that the decreased risk of the polymorphism to gastric cancer was maintained in the subgroup of male, and those with age older than 64 years, suggesting the susceptibility of the polymorphism to gastric cancer risk could be effected by demographic characteristics of participants. Due to the limited sample sized of this study, the significant should be verified by further study. *TGFBR1* rs334348 located in the 3′ UTR region, and it was suggested with location in miRNA-628-5p binding site, resulting in GG genotype turn to be associated with lower *TGFBR1* expression [24]. In addition, previous study has also reported that it could confer an increased risk of colorectal cancer by affecting *TGFBR1* expression [25].

In the retrospective study, we observed *TLR4* rs1927911 and *TGFBR1* rs10512263 were associated with clinical outcomes of gastric cancer patients. *TLR4* rs1927911 is an intron variation that was previously reported as a protective factor for gastric cancer [26, 27]; however, we failed to find such a significant association but we observed it was associated with unfavorable OS of gastric cancer patients, especially for male, patients with age younger than 64 years old, or patients with NCGC. To date, the function of rs1927911 remains unclear, we speculated that such a significant association was related the microenvironment of cancer by that TLR4 signaling was involved in drug resistant by inducing the M1 phenotype macrophages [28] and by that TLR4/NF-κB signal pathway mediated uncontrolled inflammation [29]. Moreover, this study observed *TGFBR1* rs10512263 has a predictive value for clinical outcomes of gastric cancer patients. Although the function of rs10512263 remains unclear, TGF-β signaling has been suggested to promote gastric cancer progression by enhancing motility and inducing invasiveness of gastric cancer cell [11], or by promoting tumor vasculature conformation [30], which could be partly explained for the predictive role of *TGFBR1* rs10512263 in gastric cancer patients.

Polymorphisms in three immune related genes was discussed for their susceptibility and predictive role in gastric cancer. Here, some limitations of this study should be noted. Firstly, the function of these polymorphisms is largely unclear, and we failed to assess the association of polymorphism and TGFBR1, TLR4 expression in patients. Instead of that, to perform functional candidate polymorphism and expression quantitative trait locus (eQTL) analyses on the promising genes, we mined the data from the following databases: GTExPortal (https://www.gtexportal.org/home/) and Haploreg (http://www.broadinstitute.org/mammals/haploreg/haploreg.php), and the results shown that *TLR4* rs1927911, *TGF*-*BR1* rs6478974 and rs334348 could affect their corresponding gene expression, and that *TGF*-*BR1* rs10512263 could regulate certain motifs, which were consistent to our results, see Additional file 2: Figures S1 and S2. Secondly, the sample size of this study was not large enough, which may weaken the statistical power. Thirdly, environmental factors, such as diet, physical exercises, gastric diseases history, and subtype of *H. pylori* were not included in this study, which may influence the conclusion. Finally, there are number of polymorphisms in the immune related genes, here we selected three of them and some more immune related genes required to be discussed.

## Conclusion

We concluded that two polymorphisms (rs334348, rs10512263) in *TGF*-*BR1* were associated with risk of gastric cancer, and that *TLR4* rs1927911 and *TGFBR1* rs10512263 were associated with clinical outcomes of gastric cancer patients. This is a study firstly discussed the relation of polymorphisms in genes of *IL*-*16*, *TGFBR1* and *TLR4* pathways and survival time of gastric cancer patients in Chinese population and our study could provide epidemiology data for further study.

## Additional files

**Additional file 1: Table S1.** Information of enrolled genetic variations. **Table S2.** Clinical and demographic characteristics of enrolled participants.

**Additional file 2: Figure S1.** eQTL analysis of mRNA expression in whole blood and genotype data. A: *TLR4* rs1927911, p-value = 0.000016; B: *TGF-BR1* rs6478974, p-value = 6.5e-7, and C: rs334348, p-value = 0.0000029. **Figure S2.** Results from the Haploreg website for the *TGF-BR1* rs10512263.

## Abbreviations

TGF-β1: transforming growth factor beta-1
TGFBR1: TGF-β receptor 1
*H. pylori*: *Helicobacter pylori*
TLRs: toll-like receptors
LPS: lipopolysaccharide
MAF: minor allele frequency
5′ UTR: 5′ untranslated regions
OR: odds ratios
CI: confidence intervals
HR: hazard ratios
HWE: Hardy–Weinberg equilibrium
NCGC: non-cardiac gastric cancer
