# Supplementary material for: Polymorphisms of TGFBR1, TLR4 are associated with prognosis of gastric cancer in a Chinese population
Source: Cancer Cell Int. 2018 Nov 20;18:191. doi: 10.1186/s12935-018-0682-0 (PMC6245525; doi:10.1186/s12935-018-0682-0)
Supplement: Supplementary file 1 — Additional file 1: Table S1. Information of enrolled genetic variations. Table S2. Clinical and demographic characteristics of enrolled participants. [file 12935_2018_682_MOESM1_ESM.docx]

Table S1 Information of enrolled genetic variations.

| Gene | SNP ID | Chromosome position | Allele | Position | HWE |
| --- | --- | --- | --- | --- | --- |
| *IL16* | rs4072111 | 15：81285798 | C/T | Ex6, Ser434Pro | 0.206 |
| *IL16* | rs4778889 | 15：81296654 | T/C | Promoter, -295T>C | 0.198 |
| *IL16* | rs11556218 | 15：81305928 | T/G | Ex6, Asn179Lys | 0.716 |
| *IL16* | rs859 | 15：81308981 | G/A | 3’UTR | 0.543 |
| *IL16* | rs1131445 | 15：81309441 | T/C | 3’UTR | 0.496 |
| *TLR4* | rs10759932 | 9：117702866 | T/C | Promoter, -1607T>C | 0.79 |
| *TLR4* | rs1927911 | 9：117707776 | G/A | 3’UTR | 0.536 |
| *TLR4* | rs11536889 | 9：117715853 | G/C | 3’UTR | 0.938 |
| *TGF-BR1* | rs6478974 | 9：99112121 | T/A | Intron | 0.603 |
| *TGF-BR1* | rs10512263 | 9：99123789 | T/C | Intron | 0.936 |
| *TGF-BR1* | rs334348 | 9：99150189 | A/G | 3’UTR | 0.708 |

MAF: Minor allele frequency; 5’FR: 5'flankingregion; 3’UTR: 3’ Untranslated Regions; Ex: exon; HWE: Hardy-Weinberg equilibrium

Table S2 Clinical and demographic characteristics of enrolled participants

| Variables | Cases, n (%) | Controls, n (%) | P-value |
| --- | --- | --- | --- |
| All subjects | 479 | 483 |  |
| Age (Mean±SD) | 64.48±11.91 | 64.73±11.84 | 0.748 |
| Gender |  |  |  |
| Male | 353(73.70) | 358(74.12) | 0.881 |
| Female | 126(26.30) | 125(25.88) |  |
| *H.polyri* infection |  |  |  |
| Positive | 261(54.49) | 231(47.83) | 0.039 |
| Negative | 218(45.51) | 252(52.17) |  |
| Cigarette smoking |  |  |  |
| Never | 368(76.83) | 419(86.75) | ＜0.001 |
| Ever | 111(23.17) | 64(13.25) |  |
| Alcohol consumption |  |  |  |
| No | 426(88.94) | 461(95.45) | ＜0.001 |
| Yes | 53(11.06) | 22(4.55) |  |
| TNM stages |  |  |  |
| Ⅰ-Ⅱ | 159(33.19) |  |  |
| Ⅲ-Ⅳ | 320(66.81) |  |  |
| Tumor site |  |  |  |
| Cardia | 138(28.81) |  |  |
| Non-cardia | 341(71.19) |  |  |
