# Supplementary material for: Polymorphisms of TGFBR1, TLR4 are associated with prognosis of gastric cancer in a Chinese population
Source: Cancer Cell Int. 2018 Nov 20;18:191. doi: 10.1186/s12935-018-0682-0 (PMC6245525; doi:10.1186/s12935-018-0682-0)
Supplement: Supplementary file 2 — Additional file 2: Figure S1. eQTL analysis of mRNA expression in whole blood and genotype data. A: TLR4 rs1927911, p-value = 0.000016; B: TGF-BR1 rs6478974, p-value = 6.5e-7, and C: rs334348, p-value = 0.0000029. Figure S2. Results from the Haploreg website for the TGF-BR1 rs10512263. [file 12935_2018_682_MOESM2_ESM.pdf]

Additional file 2 Figure S1.

eQTL analysis of mRNA expression in whole blood and genotype data. A: *TLR4* rs1927911, p-value=0.000016*;* B: *TGF-BR1* rs6478974, p-value=6.5e-7, and C: rs334348, p-value= 0.0000029.

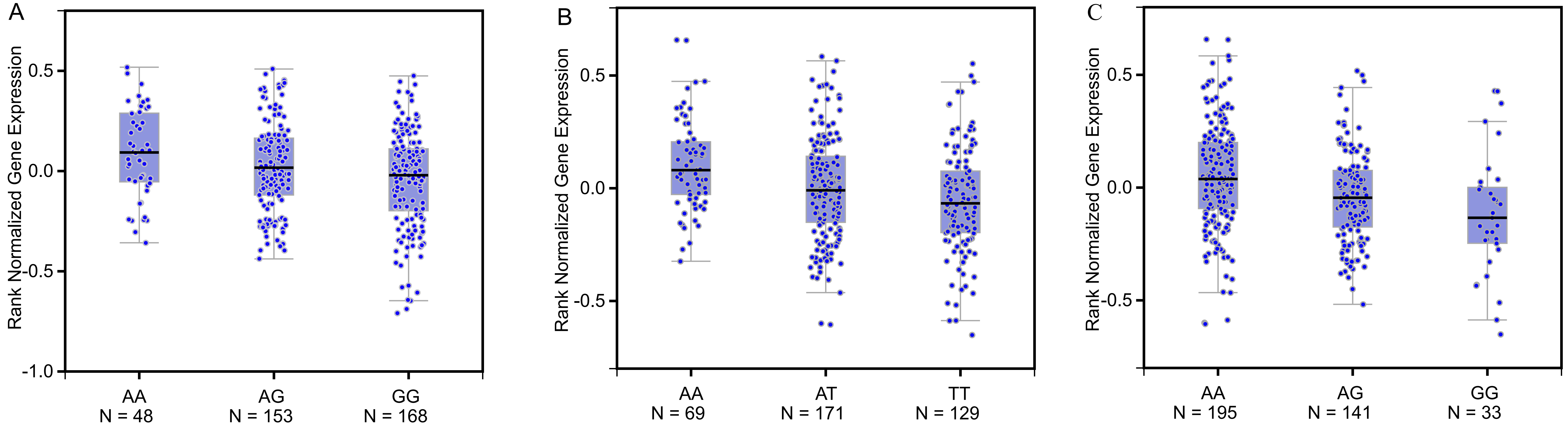
A
Rank Normalized Gene Expression
0.5
0.0
-0.5
-1.0
AA
N = 48
AG
N = 153
GG
N = 168
B
Rank Normalized Gene Expression
0.5
0.0
-0.5
AA
N = 69
AT
N = 171
TT
N = 129
C
Rank Normalized Gene Expression
0.5
0.0
-0.5
AA
N = 195
AG
N = 141
GG
N = 33

Additional file 2 Figure S2. Results from the Haploreg website for the *TGF-BR1* rs10512263

Query SNP: rs10512263 and variants with r^2 >= 0.8

chr	pos (hg38)	LD (r^2)	LD (D')	variant	Ref	Alt	AFR freq	AMR freq	ASN freq	EUR freq	SiPhy cons	Promoter histone marks	Enhancer histone marks	DNAse	Proteins bound	Motifs changed	NHGRI/EBI GWAS hits	GRASP QTL hits	Selected eQTL hits	GENCODE genes	dbSNP func annot
9	99123789	1	1	rs10512263	T	C	0.02	0.11	0.26	0.07				BLD		Ik-1,STAT				TGFBR1	intronic

Regulatory motifs altered

Position Weight Matrix ID (Library from Kheradpour and Kellis, 2013)	Strand	Ref	Alt	Match on:<br>Ref: ATTCTACAGTGGAATTTTGTCACTACTTTTGGGAAGAAGTTTACCTTTGGTGGCCTACG<br>Alt: ATTCTACAGTGGAATTTTGTCACTACTTTCGGGAAGAAGTTTACCTTTGGTGGCCTACG
Ik-1_2	+	12.9	9.8	KYTRGGRRR
STAT_known10	-	-5.5	6.4	BDBVAHTTCCSGGAADTRRSR
